# A Transcriptomic Signature of the Hypothalamic Response to Fasting and BDNF Deficiency in Prader-Willi Syndrome

**DOI:** 10.1016/j.celrep.2018.03.018

**Published:** 2018-03-05

**Authors:** Elena G. Bochukova, Katherine Lawler, Sophie Croizier, Julia M. Keogh, Nisha Patel, Garth Strohbehn, Kitty K. Lo, Jack Humphrey, Anita Hokken-Koelega, Layla Damen, Stephany Donze, Sebastien G. Bouret, Vincent Plagnol, I. Sadaf Farooqi

**Affiliations:** 1 University of Cambridge Metabolic Research Laboratories and NIHR Cambridge Biomedical Research Centre, Wellcome Trust-MRC Institute of Metabolic Science, Addenbrooke’s Hospital, Cambridge CB2 0QQ, UK; 2 The Blizard Institute, Barts and The London School of Medicine and Dentistry, Queen Mary University of London, London E1 2AT, UK; 3 The Saban Research Institute, Developmental Neuroscience Program, and Diabetes and Obesity Program, Children’s Hospital Los Angeles, Center for Endocrinology, Diabetes and Metabolism, University of Southern California, Los Angeles, CA 90027, USA; 4 Inserm, Jean-Pierre Aubert Research Center, U1172, University Lille 2, Lille, 59045, France; 5 Center for Integrative Genomics, University of Lausanne, Lausanne, Switzerland; 6 University College London Genetics Institute (UGI), Department of Genetics, Environment and Evolution, University College London, Darwin Building, Gower Street, London, WC1E 6BT, UK; 7 Department of Neurodegenerative Disease, University College London Institute of Neurology, London, WC1N 3BG, UK; 8 Erasmus University Medical Center, Rotterdam, the Netherlands; 9 Dutch Growth Research Foundation, Rotterdam, the Netherlands

**Keywords:** hypothalamus, Prader-Willi syndrome, BDNF, Agrp, obesity, SNORD116

## Abstract

Transcriptional analysis of brain tissue from people with molecularly defined causes of obesity may highlight disease mechanisms and therapeutic targets. We performed RNA sequencing of hypothalamus from individuals with Prader-Willi syndrome (PWS), a genetic obesity syndrome characterized by severe hyperphagia. We found that upregulated genes overlap with the transcriptome of mouse Agrp neurons that signal hunger, while downregulated genes overlap with the expression profile of Pomc neurons activated by feeding. Downregulated genes are expressed mainly in neuronal cells and contribute to neurogenesis, neurotransmitter release, and synaptic plasticity, while upregulated, predominantly microglial genes are involved in inflammatory responses. This transcriptional signature may be mediated by reduced brain-derived neurotrophic factor expression. Additionally, we implicate disruption of alternative splicing as a potential molecular mechanism underlying neuronal dysfunction in PWS. Transcriptomic analysis of the human hypothalamus may identify neural mechanisms involved in energy homeostasis and potential therapeutic targets for weight loss.

## Highlights

• Overlap between genes expressed in human PWS hypothalamus and mouse Agrp neurons
• Downregulated genes are involved in neuronal development
• SNORD116 deletion reduces neural development and survival in cells
• Alternative splicing is disturbed in PWS

## Graphical Abstract

**Figure:**
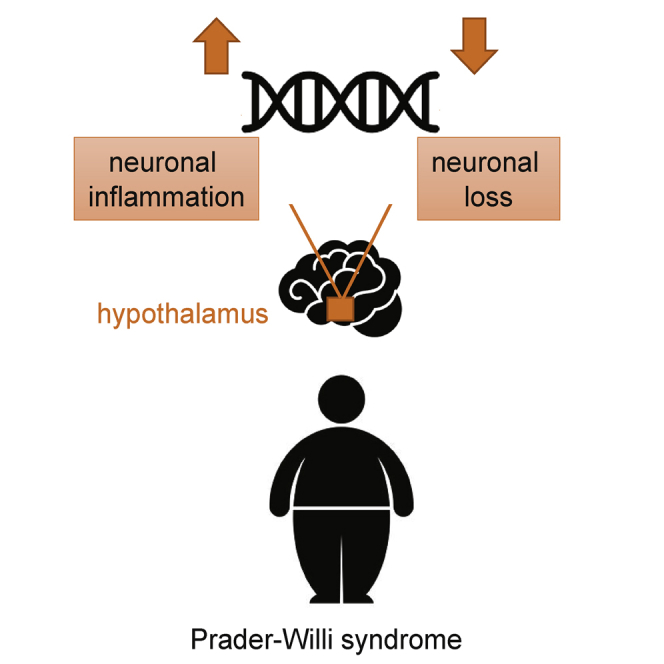

## Teaser

Prader-Willi syndrome (PWS) is a genetic obesity syndrome. Bochukova et al. report gene expression changes in the hypothalamus of people with PWS that support neurodegeneration and neuroinflammation as key processes involved in this condition.

## Introduction

Neural circuits within the hypothalamus regulate energy balance in response to peripheral nutrient-related cues (Andermann and Lowell, 2017, Gautron et al., 2015). Leptin-responsive Agouti-related protein (Agrp)-expressing neurons in the arcuate nucleus of the hypothalamus are activated during fasting or caloric deficit to drive an increase in food intake, while in the nutritionally replete or fed state, Pro-opiomelanocortin (Pomc) neurons are activated to reduce food intake (Cowley et al., 1999, Cowley et al., 2001). In humans, loss-of-function mutations that disrupt the function of these neural circuits result in severe obesity, demonstrating their pivotal role in human energy homeostasis (O’Rahilly and Farooqi, 2008, van der Klaauw and Farooqi, 2015).

However, experiments in rodents (Atasoy et al., 2012, Betley et al., 2013) and genetic studies in humans (Hendricks et al., 2017) suggest that the neural mechanisms that regulate energy homeostasis are complex and that many molecular components of these circuits remain to be discovered (Sternson et al., 2016). One potential approach to identifying genes and pathways is to use transcriptomic analysis of key tissues and organs to identify changes in gene expression in response to a perturbation or genetic manipulation. The specificity of these approaches has been enhanced by recent technological developments that have enabled the labeling, sorting, and RNA sequencing of molecularly defined populations of neurons in the mouse brain. To this end, the recent detailed analysis of high-quality gene expression data from mouse Agrp and Pomc neurons has provided a framework for investigating the genes whose expression changes with fasting and feeding (Campbell et al., 2017, Henry et al., 2015). Although comparable studies of specific cell types are not feasible in humans, transcriptional analysis of hypothalamic tissue from people with molecularly defined subtypes of severe obesity has the potential to inform the discovery of neural mechanisms involved in energy balance. Here, we characterized the hypothalamic transcriptome of individuals with Prader-Willi syndrome (PWS), a genetic obesity syndrome caused by loss of expression of paternally expressed genes and noncoding RNAs on chromosome 15q11–q13 (Cassidy et al., 2012).

## Results and Discussion

**Figure 1:**
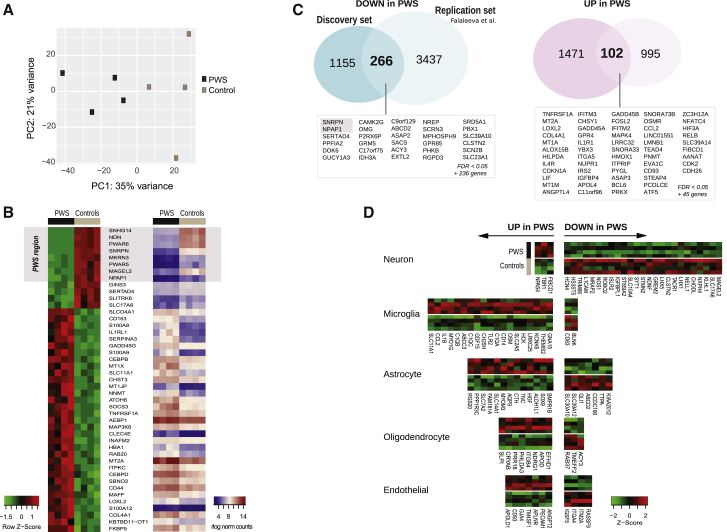
Genome-wide Transcriptional Changes in PWS Hypothalamus (A) Principal-component (PC) analysis showing segregation of PWS and control hypothalamic samples. (B) Heatmap representing the top 45 most significantly DEGs shown as within-gene *Z* score (left) and rlog-normalized read counts (right). (C) Venn diagrams illustrating differentially down- and upregulated genes in PWS versus control samples in this study (discovery set) and overlap with genes from a previous study in PWS (replication set) (Falaleeva et al., 2015). (D) Heatmaps representing the expression of brain cell-type-specific genes among the DEGs displayed as within-gene *Z* score of rlog-normalized read counts. See also Figure S1 and Table S1.

RNA sequencing was performed on post-mortem hypothalamic tissue from four PWS patients and four age-matched controls from the University of Maryland Brain and Tissue Bank (Figure S1). Although samples from controls matched for both age and obesity were not available, the body mass index (BMI) values of patients and controls were comparable (Figure S1A). Principal-component analysis revealed segregation between PWS and control samples (Figure 1A). We identified 3,676 differentially expressed genes (DEGs) in PWS individuals compared with controls (Table S1; Benjamini-Hochberg false discovery rate [FDR] < 0.25; 658 with FDR < 0.05). The most highly downregulated genes (FDR < 5 × 10^−5^) were located in the PWS critical region (Figure 1B). A random subset of genes were validated by qRT-PCR (Figure S1E). In the absence of high-quality hypothalamic tissue for replication, we compared our data with a previous high-density microarray study of hypothalamic gene expression in two PWS patients (Falaleeva et al., 2015) and found significant overlap of dysregulated genes (Figures 1C and S1D; Table S1). However, there was minimal overlap with datasets derived from PWS induced pluripotent stem cell (iPSC)-derived neuronal cell lines (data not shown); notably, we did not find reduced expression of the obesity-associated gene PCSK1 reported recently (Burnett et al., 2017b).

To identify the cellular origin of DEGs, we ranked genes on the basis of their relative expression in single-cell transcriptomic data from neurons, astrocytes, microglia, oligodendrocytes, and endothelial cells (Supplemental Experimental Procedures). We found that downregulated genes were enriched for neuronal markers (p = 3 × 10^−8^), while upregulated genes were enriched for microglial genes (p = 9 × 10^−5^) (Figure 1D). Further analysis using CIBERSORT (Newman et al., 2015) also showed that PWS hypothalamic tissue was characterized by a reduction in neurons (Figure S1F). Interestingly, this cellular transcriptomic profile aligns with that seen in autism (Parikshak et al., 2016), in several neurodegenerative diseases, and in the aging brain (Blalock et al., 2004, Lu et al., 2004) (Figure S2A), suggesting that fundamental mechanisms regulating neuronal maintenance may contribute to a range of human neurological diseases, including PWS.

### Overlap of the Human PWS Transcriptome with the Transcriptome of Agrp Neurons in Fasting

**Figure 2:**
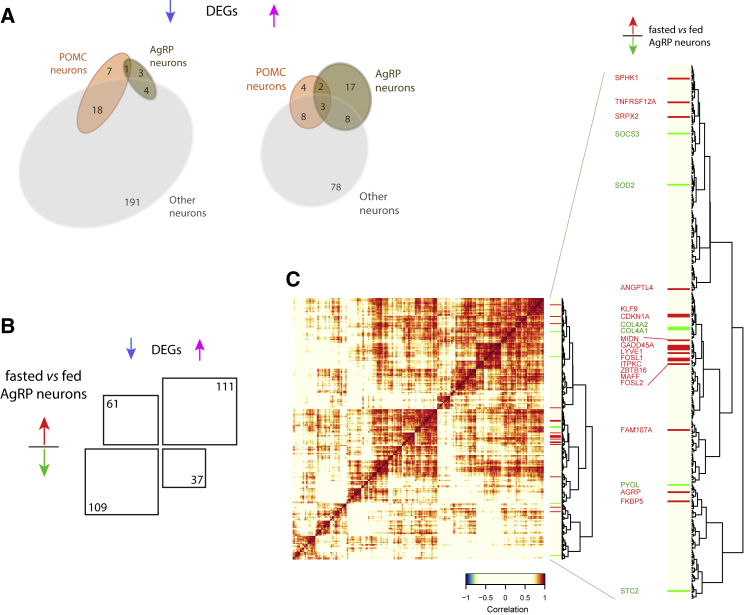
Dysregulated Gene Co-expression Modules in PWS Hypothalamus Converge with Fasting and Feeding Responses in Specific Hypothalamic Cell Types from Mice (A) Venn diagrams illustrating the number of DEGs that are down- and upregulated in PWS hypothalami compared with controls and their expression in Pomc, Agrp, and other neurons (Campbell et al., 2017, Henry et al., 2015). For comparison, the reference gene sets (Pomc, 261 genes; Agrp, 167 genes; other neurons, 1,589 genes) are included in Figure S2A. (B) Number of PWS DEGs (up- or downregulated) that are expressed in Agrp neurons in the fasted versus fed state (q < 0.05 in Henry et al., 2015). (C) Gene co-expression modules among upregulated PWS DEGs. Hierarchical clustering of DEGs upregulated in PWS with log_2_ fold change >1.5. The heatmap illustrates pairwise gene-gene correlation clustering (Pearson correlation, distance = 1-cor, Ward clustering). The sidebar (right) displays the overlap with genes previously reported upregulated (red) or downregulated (green) in Agrp neurons in the fasted versus fed state (q < 0.05 in Henry et al., 2015). See also Figure S2 and Table S1.

To identify potential candidate obesity genes, we compared PWS DEGs with genes expressed in hypothalamic Agrp and Pomc neurons in mice (Campbell et al., 2017, Henry et al., 2015) (Supplemental Experimental Procedures). We found that expression of Agrp was increased 3-fold in PWS hypothalamus versus controls (p = 0.01), suggesting this potent orexigenic may play a role in the hyperphagia associated with PWS. Other upregulated genes were predominantly expressed in mouse Agrp neurons that signal hunger, while genes downregulated in PWS were relatively overrepresented in mouse Pomc neurons that signal the fed state (Fisher’s exact test, odds ratio [OR] = 7.2, p = 2.3 × 10^−4^) (Figures 2A and S2). A significant number of PWS upregulated genes were expressed in mouse Agrp neurons and upregulated in fasted animals (Fisher’s exact test, OR = 5.3, p = 10^−12^; Figure 2B), suggesting that these genes represent a conserved signature of the neural response to fasting or food deprivation.

Using hierarchical cluster analyses of high-confidence DEGs (absolute log fold change > 1.5), we identified sets of co-expressed genes and gene modules whose expression was upregulated in Agrp neurons in the fasted state (Figure 2C). We observed increased expression of ribosomal proteins involved in protein synthesis. This finding aligns with the upregulation of genes involved in endoplasmic reticulum (ER) protein translocation and Golgi trafficking seen in Agrp neurons in mice with fasting (Henry et al., 2015) and may reflect increased production of neuropeptides for secretion. Several genes downregulated in PWS, and also in mouse Pomc neurons, were involved in synaptic transmission and neuronal maintenance and integrity. As loss-of-function mutations in some of these genes (SRPX2 and ZBTB16; Table S1) are known to cause human neurological disorders, their reduced expression could contribute to both the obesity and the neurodevelopmental phenotype of PWS.

A subset of co-regulated genes dysregulated in the PWS hypothalamus are expressed in Agrp neurons in fasting and are known to play a role in energy homeostasis and adipocyte biology in rodents (SOCS3, ANGPTL4, FOSL1, FOSL2, and STC2; Table S1). Interestingly, bone morphogenic factor-3 (BMP3), whose expression is markedly decreased in mouse Agrp neurons in the fasted state (−17.7-fold, q = 2.0 × 10^−5^; Henry et al., 2015), was found to be significantly decreased in the human PWS hypothalamus. These findings generate hypotheses that will need to be explored further. Characterization of the neurons in which these genes are expressed and the processes they regulate, as well as DEGs expressed in other transcriptionally distinct neuronal cell types, may provide insights into the mechanisms involved in human energy balance.

### Human PWS Hypothalamus Is Characterized by Downregulation of Genes Involved in Neuronal Function and Upregulation of Microglial Genes and Inflammatory Markers

**Figure 3:**
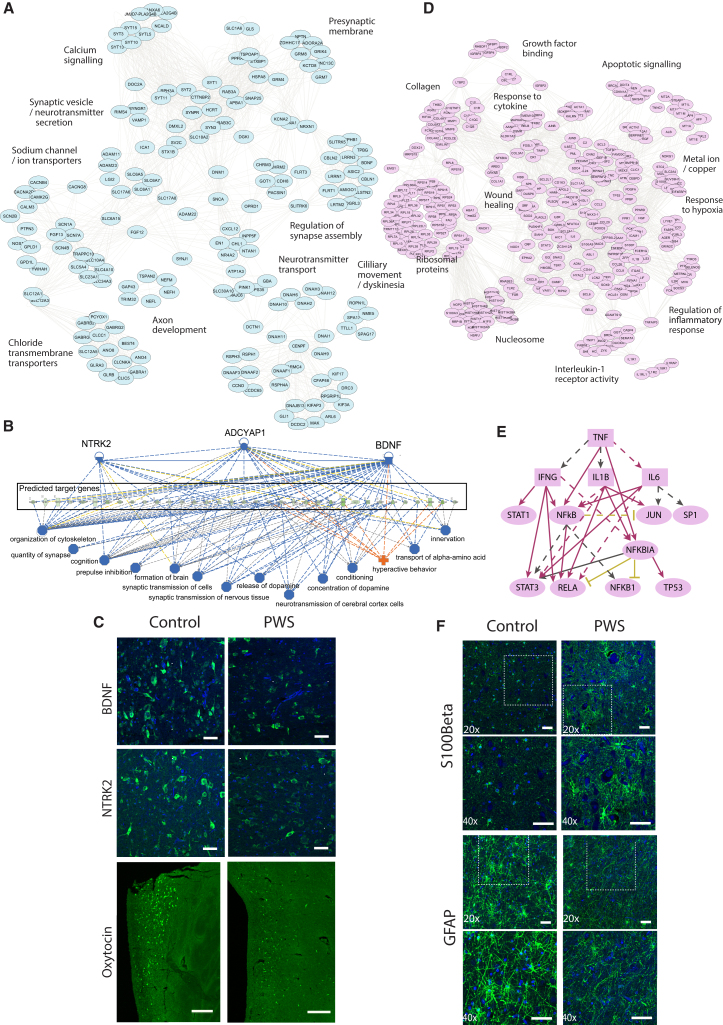
Pathways Predicted to Be Affected by Changes in Gene Expression Seen in PWS Hypothalamus (A) A gene annotation network illustrating terms (Gene Ontology, Reactome, Key) enriched among downregulated DEGs. Nodes represent downregulated DEGs annotated with illustrated terms; edges join pairs of genes annotated with the respective term. (B) Ingenuity Pathway Analysis (IPA) regulator effects analysis indicates the inhibition of regulatory factors NTRK2, ADCYAP1, and BDNF (top) with predicted effects on target genes and processes. Phenotypes predicted to occur as a consequence of the gene expression changes are shown in blue (inhibited) or orange (enhanced). (C) Representative FISH images of BDNF and NTRK2 mRNA-expressing cells in the ventromedial nucleus of the hypothalamus and oxytocin mRNA-expressing cells in the paraventricular nucleus of the hypothalamus in PWS and control samples (BDNF [n = 2 PWS, n = 2 controls], NTRK2 [n = 2 PWS, n = 1 control], and oxytocin [n = 2 PWS, n = 1 control]). (D) A gene annotation network illustrating terms enriched among upregulated DEGs. Nodes and edges as in Figure 2A. (E) IPA upstream regulator analysis indicates inhibition of TNF/NFKb signaling. (F) Representative immunohistochemistry images of S100Beta- and GFAP-immunoreactive cells in the ventromedial nucleus of the hypothalamus in PWS and control samples. See also Figure S3 and Table S2.

We found that downregulated DEGs were significantly enriched for genes involved in certain processes, namely, neurogenesis, neurotransmitter release, and synaptic function (Figure 3A). Using Ingenuity Pathway Analysis, we identified 11 potential regulators of clusters of downregulated DEGs (Table S2), including the neurotrophin brain-derived neurotrophic factor (BDNF) and its receptor, TrkB (encoded by NTRK2). Putative BDNF/TrkB targets among the downregulated DEGs were predominantly related to synaptic processes (Figure 3B). This finding is intriguing, as BDNF is a major regulator of the development, maturation, and maintenance of neurons and a modulator of synaptic plasticity (Snider, 1994). Moreover, in mice and humans, genetic disruption of BDNF and TrkB causes developmental delay, stereotyped behaviors, impaired pain sensation, hyperphagia, and severe obesity (Gray et al., 2006, Yeo et al., 2004), phenotypes that show some overlap with those seen in PWS. We also obtained several post-mortem brain samples for histology. Very few samples were of sufficient quality, limiting quantitative analysis, but fluorescence *in situ* hybridization of human hypothalamic tissue suggested that the number of cells expressing BDNF and NTRK2 mRNA was reduced in the ventromedial nucleus of the hypothalamus in PWS (Figures 3C and S3). We measured levels of plasma BDNF (potentially derived from platelets) in patients with PWS versus age-matched obese controls, but we did not find a significant difference (Figure S3G), in contrast to one previous study (Han et al., 2010). Possible explanations are that BDNF levels are known to vary considerably in plasma versus serum and among assays; additionally, plasma BDNF may not reflect BDNF expression in the brain.

A previous histopathological study of the PWS hypothalamus found a significantly reduced number of oxytocin neurons (Swaab et al., 1995), and clinical trials of intranasal oxytocin administration in PWS are ongoing (Tauber et al., 2017). In our study, we found a low level of oxytocin mRNA and a smaller number of cells immunoreactive for oxytocin in the paraventricular nucleus in PWS samples (Figure 3C), supporting the key role of oxytocin as well as BDNF in the neuropathology of PWS. Additional studies are needed to replicate these findings and to investigate the potential loss of other neuronal populations (including Pomc and Agrp neurons) within the hypothalamus in PWS.

We found that upregulated genes in the PWS hypothalamus were enriched for cytokine signaling and inflammatory processes (Figure 3D; Table S2). The most statistically significant predicted regulator of these genes was tumor necrosis factor (TNF)-alpha, which plays a critical role in systemic inflammation (Figure 3E; Table S2). In the human hypothalamus, we studied expression of S100b (a glial-specific protein marker of neural damage) and GFAP (an astrocyte filament protein that plays a critical role in synaptic function and is reduced in neurodegenerative disorders but increased in brain injury). We found that S100b protein levels were increased and GFAP immunoreactivity was decreased in the PWS hypothalamus compared with controls (Figure 3F). These findings overlap with data from other neurodevelopmental conditions (Griffin et al., 1989). Further studies with larger sample sizes are needed to explore the potential relevance of these findings.

### Targeted Deletion of SNORD116 Affects Neuronal Differentiation, Proliferation, and Survival

**Figure 4:**
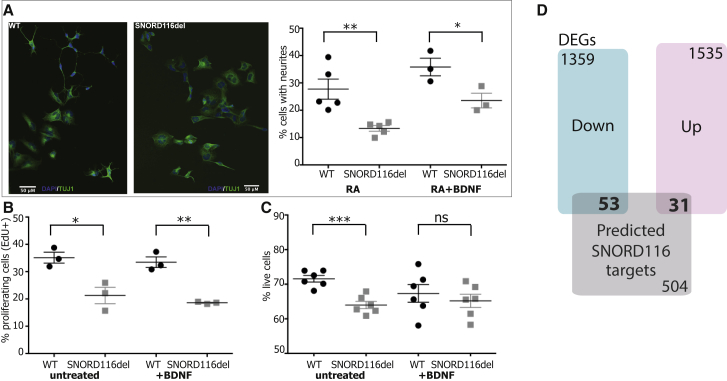
Deletion of SNORD116 Impairs Neuronal Differentiation, Proliferation, and Survival (A) Targeted deletion of SNORD116 (SNORD116del) affects the neuronal differentiation of SH-SY5Y cells, cultured for 7 days in retinoic acid (RA) in the absence (n = 5) or presence (n = 3) of BDNF. Left: representative images of wild-type (WT) and SNORD116del cells; right: quantification plot. (B) Cellular proliferation measured by EdU incorporation at day 7 (n = 3). (C) Cell survival measured by FACS at day 7 in culture (n = 6). (D) Overlap between *in silico* predicted SNORD116 gene targets and PWS differentially expressed and differentially spliced genes. All data are presented as mean ± SEM. Statistical significance was measured using two-tailed Student’s t test (^∗^p < 0.05, ^∗∗^p < 0.01, ^∗∗∗^p < 0.001; ns, non-significance). See also Figure S4 and Tables S3 and S4.

Chromosomal deletions that cause PWS vary in size and thus can affect a number of genes and noncoding RNAs. None of the mouse models involving deletion of the homologous region fully recapitulate the human PWS phenotype (Resnick et al., 2013); as such, investigation of the molecular mechanisms that underlie the clinical phenotype has been challenging. The minimal genetic lesion associated with severe hyperphagia and obesity in PWS contains a cluster of noncoding small nucleolar RNAs (snoRNAs) referred to as the SNORD116 gene cluster (de Smith et al., 2009, Sahoo et al., 2008). Postnatal deletion of SNORD116 in the mediobasal hypothalamus has recently been shown to lead to increased food intake in mice (Polex-Wolf et al., 2018). To test whether loss of SNORD116 affects neuronal development and maintenance, as suggested by our transcriptomics analysis and in line with a rodent model (Burnett et al., 2017a), we deleted a 57.4 kb genomic segment encompassing the SNORD116 cluster using CRISPR-Cas9 in a SH-SY5Y neuroblastoma human cell line (Figure S4A). We found that SNORD116-deficient cells exhibited reduced neuronal differentiation, cell proliferation, and survival compared with wild-type cells (Figures 4A–4C). A higher proportion of SNORD116-deficient cells displayed neurites when treated with BDNF (mean 13%) compared with no treatment (mean 23%, p = 0.005, two-tailed t test), whereas no significant difference was observed within wild-type cells (28% with no treatment, 36% with BDNF; p = 0.2, two-tailed t test). Cumulatively, these data identify a transcriptomic signature in PWS consistent with marked hypothalamic neurodegeneration, which may be mediated in part by reduced expression of the neurotrophin BDNF and its receptor, TrkB. These data align with experiments in cortical neurons of the SNORD116 knockout mouse (Burnett et al., 2017a). Neuronal loss is associated with a marked inflammatory response in the hypothalamus, which may be a primary defect, secondary to the neurodegenerative process or, as microglia have a role in synaptic development and function (Barres, 2008), an inflammatory response to disordered synaptic plasticity in the PWS hypothalamus.

### Predicted snoRNA Targets and Detection of Reduced Splicing Efficiency

SNORD116 and the closely related SNORD115 cluster belong to a group of orphan snoRNAs with presumed non-canonical functions. SNORD115 has been shown to regulate the post-transcriptional processing of a single pre-mRNA, the serotonin 2c receptor, through alternative splicing and RNA editing (Kishore and Stamm, 2006). Using snoTARGET, we identified 588 predicted targets for snoRNAs within protein-coding genes (Figure 4D; Table S3), some of which were differentially expressed in PWS hypothalamus (Figure S4B). Further studies will be needed to test the functional significance of these findings. Interestingly, RNA-specific adenosine deaminase (ADARB1), a predicted target that is significantly downregulated (Figure S4B), is involved in pre-mRNA editing of glutamate receptor subunit B and when deleted causes hyperphagia and obesity in mice (Terajima et al., 2017).

As snoRNAs can modulate RNA splicing (Yin et al., 2012), a process that plays a major role in human neuronal development, we performed a transcriptome-wide search for evidence of alternative splicing (Supplemental Experimental Procedures). We found evidence of differential use of alternative splice variants in PWS samples compared with controls (Table S4). Focusing on 180 loci with evidence of differential use of two alternative splice variants, the most frequently observed type of splice variant in PWS was retained introns (Table S4; Figure S4). Of note, we did not find evidence for differential splicing of the serotonin 2c receptor (Figure S4C). Genes with putative differential splicing did not tend to be differentially expressed, consistent with decoupling of differential expression and splicing as seen in other disorders; exceptions included genes involved in microglial and inflammatory processes, which were among the top-ranked alternatively spliced genes (Table S4). Motif searches within retained introns and 250 bp flanking regions indicated the presence of binding sites for canonical serine/arginine-rich splicing factors, and the presence of binding sites with predicted similarity to FUS splicing factor binding motifs (Figure S4E). The FUS splicing factor regulates alternative splicing in the brain and has been previously linked to neurodegenerative diseases including amyotrophic lateral sclerosis (ALS) and frontotemporal lobar degeneration (FTLD) (Ishigaki et al., 2012, Rogelj et al., 2012).

In summary, in this study of the human hypothalamus in a small number of individuals with PWS, we identified a transcriptomic signature characterized by neuronal loss, altered neuro-plasticity, and neuroinflammation. Of note, several neuroimaging studies and case reports in PWS have identified structural abnormalities that would be consistent with a reduced number of neurons, such as reduced gray matter volume in a number of cortical areas and abnormal gyrification (Manning and Holland, 2015). We identify a potential role for BDNF in PWS that requires further exploration and may have therapeutic relevance for this complex neuro-behavioral disorder. Additionally, we demonstrate that transcriptomic analysis of the human hypothalamus can generate testable hypotheses of potential relevance to the understanding of the neural circuits involved in human energy homeostasis.

## Experimental Procedures

### Human Samples

Hypothalamic specimens used in the study were obtained at autopsy from control subjects with no reported clinical signs and patients with genetic diagnoses of PWS through the University of Maryland Brain Bank at the University of Maryland (Figure S1A). All procedures were approved by the University of Cambridge Human Biology Research Ethics Committee (HBREC.2014.14).

### RNA Sequencing and Analysis

Total RNA was prepared by tissue homogenization in Trizol reagent (Thermo Fisher Scientific) of about one-third of hypothalamus. Sequencing of RNA samples was performed by the University College London (UCL) Genomics core facility, using the TruSeq poly-A mRNA method (Illumina) and a HiSeq 2000 machine (Illumina). Differential expression, splicing, and pathway analysis are described in detail in Supplemental Experimental Procedures, as is the validation of DEGs using qRT-PCR.

### *In Silico* Prediction of SNORD116 Gene Targets

Genome-wide *in silico* prediction of SNORD116 targets was performed using snoTARGET software (Bazeley et al., 2008) and RNA-cofold from the Vienna RNA package (http://www.tbi.univie.ac.at/RNA/).

### Cross-Species Comparison with Agrp and Pomc Neuronal Subtypes and Response to Food Deprivation

Reference gene sets for broad neuronal subtype classifications were derived from Campbell et al. (2017) as described in Supplemental Experimental Procedures. Reference gene sets for fasting response in Agrp neurons were obtained from Henry et al. (2015) using a threshold of q < 0.05 (unless otherwise stated) to define differential expression between fasting conditions.

### Immunohistochemistry and Fluorescence *In Situ* Hybridization

Immunohistochemistry was performed as reported previously (Bouret et al., 2004) using the following primary antibodies: guinea pig anti-oxytocin (Peninsula Laboratories), rabbit anti-GFAP (Dako), and rabbit anti-s100beta (Abcam). Secondary antibodies were Alexa Fluor 488 donkey anti-guinea-pig IgGs or Alexa Fluor 488 goat anti-rabbit IgGs (Thermo Fisher Scientific). For the fluorescence *in situ* hybridization (FISH) experiments, sense and antisense digoxigenin-labeled riboprobes were generated from plasmids containing PCR fragments of BDNF and NTRK2 (generously provided by Dr. Baoji Xu, The Scripps Research Institute). Staining density and cell number were calculated using ImageJ analysis software (NIH). Full details are presented in Supplemental Experimental Procedures.

### Cellular Studies

SH-SY5Y (ATCC CRL-2266) cells were used in all the cellular assays. We used a well-established protocol to differentiate SH-SY5Y cells into neurons with retinoic acid (Encinas et al., 2000). Full details on maintenance, neuronal differentiation, proliferation, and cell survival are presented in Supplemental Experimental Procedures.

### SNORD116 Cluster Deletion Using CRISPR-Cas9

We applied a cloning-free CRISPR protocol using gBlocks (gene fragments) encoding FE-modified single guide RNAs (sgRNAs) promoting enhanced stability (Arbab et al., 2015). Two gBlocks carrying the guide flanking the SNORD116 cluster on chr15q11.2 were nucleofected alongside GFP-expressing Cas9 plasmid PX458into the SH-SY5Y line. Fluorescence-activated cell sorting (FACS)-sorted cells were screened for successful editing using conventional PCR and confirmed by Sanger sequencing. Full details are presented in Supplemental Experimental Procedures.

### Statistical Analysis

Statistical analyses were performed using GraphPad Prism version 6.0 for MacOS X. Data are represented as mean ± SEM. A two-tailed Student’s unpaired t test was used, and p values < 0.05 were considered to indicate statistical significance.

## Data and Software Availability

The accession number for the RNA sequencing data reported in this paper is EGA: EGAS00001002901.

## Supplemental Information

Document S1. Supplemental Experimental Procedures and Figures S1–S4

Table S1. DEGs in PWS Patients versus Controls Displayed by Ensembl GeneID, Related to Figures 1 and 2 Grey shading indicates genes located in the PWS region. Column “ncRNA” indicates gene names for Ensembl gene biotypes lincRNA, miRNA, processed transcript, TEC (‘to be experimentally confirmed’); or overlapping miRNAs. Also shown are overlaps with Falaleeva et al. (2015) and putative function of genes among co-expressed gene clusters overlapping with Henry et al. (2015) fasted versus fed in Agrp neurons.

Table S2. IPA Upstream Regulator Analysis of Down, Up, or All DEGs, Related to Figure 3 IPA Ingenuity Upstream Regulator Analysis reporting putative upstream regulators top-ranked by “Activation z-score,” and p values (Fisher’s Exact Test) for overlaps with putative target genes (see Supplemental Experimental Procedures).

Table S3. List of Potential SNORD116 Targets Generated Using Software snoTARGET (Bazeley et al., 2008), Related to Figure 4

Table S4. Differential Splicing in PWS versus Controls, Related to Figures 4 and S4 List of differential splicing events displayed thresholded to FDR < 0.25.

Document S2. Article plus Supplemental Information
