# Supplementary material for: A Transcriptomic Signature of the Hypothalamic Response to Fasting and BDNF Deficiency in Prader-Willi Syndrome
Source: Cell Rep. 2018 Mar 5;22(13):3401–8. doi: 10.1016/j.celrep.2018.03.018 (PMC5896230; doi:10.1016/j.celrep.2018.03.018)
Supplement: Document S1. Supplemental Experimental Procedures and Figures S1–S4 [file mmc1.pdf]

Cell Reports, Volume *22*

Supplemental Information

A Transcriptomic Signature of the Hypothalamic Response to Fasting and BDNF Deficiency in Prader-Willi Syndrome

Elena G. Bochukova, Katherine Lawler, Sophie Croizier, Julia M. Keogh, Nisha Patel, Garth Strohbehn, Kitty K. Lo, Jack Humphrey, Anita Hokken-Koelega, Layla Damen, Stephany Donze, Sebastien G. Bouret, Vincent Plagnol, and I. Sadaf Farooqi

SUPPLEMENTARY EXPERIMENTAL PROCEDURES

Ethical approval and sample details

All procedures were approved by the University of Cambridge Human Biology Research Ethics Committee (HBREC.2014.14). Hypothalamic specimens used in the study were obtained at autopsy from control subjects with no reported clinical signs and cases with a genetic diagnosis of Prader-Willi syndrome through the University of Maryland Brain Bank at the University of Maryland (Figure S1A). The collection protocol is available from the University of Maryland Brain Bank:
(http://www.medschool.umaryland.edu/btbank/Brain-Protocol-Methods/Brain-Sectioning---Minimum-Protocol/). At autopsy, the left midbrain/brainstem were immediately frozen in liquid nitrogen and stored at -80°C until the hypothalamus was dissected upon sample retrieval. Tissue from four cases and four controls closely matched for age and post-mortem interval was obtained for RNA sequencing following hypothalamic dissection (Figure S1A,B). Sample metadata including age of the individual, post-mortem interval, limited peri-mortem clinical history, and post-mortem BMI were obtained from the tissue bank. There was variation in BMI across both the PWS and control groups (Figure S1A) which may be due to multiple factors, including disease-related factors such as treatment with growth hormone. In the absence of detailed clinical and treatment information, BMI was not considered a useful variable for further inspection in this study.

RNA sequencing and analysis of expression and splicing

RNA extraction, library preparation and sequencing

Total RNA was prepared by tissue homogenization in Trizol reagent (Thermo Scientific, UK) of ~1/3 of hypothalamus (enriched for lateral and posterior hypothalamus) using Lysing Matrix D columns (MP Biomedicals, Anachem UK) and FastPrep 24 (MPI Biomedicals, Anachem UK) benchtop homogenizer according to manufacturer’s instructions. The quality and purity of RNA samples was determined by Total RNA Pico Chip (Agilent Technologies, Stockport, UK) on Agilent BioAnalyzer, according to manufacturer’s instructions.

RNA sequencing

Sequencing of RNA samples was performed by UCL Genomics core facility (University College London, UK). Preparation of indexed cDNA sequencing libraries was carried out using the TruSeq poly-A mRNA method (Illumina). Briefly, poly-A mRNA transcripts were captured from total RNA using poly-T beads, before cDNA was generated using random hexamer priming. Paired-end sequencing (2 × 100 cycles) of indexed cDNA libraries was then carried out on a HiSeq 2000 machine (Illumina), generating at least 50 million reads (101 base pairs) per sample (details in Figure S1A). After sequencing the indexed samples were demultiplexed before generation of FASTQ files for analysis.

Read alignment and quality control

Reads were aligned to *hg38* using STAR (v2.4.2a) (Dobin et al., 2013). Aligned reads were sorted and duplicates marked using NovoSort (novocraft3; Novocraft, Novocraft Technologies Sdn Bhd, Malaysia). Quality control following read alignment was performed using QoRTs (v1.1.6) (Hartley and Mullikin, 2015). In agreement with other brain tissue studies (Parikshak et al., 2016) we observed a 3' bias which is likely to be related to RNA degradation, therefore our downstream analyses were designed to mitigate the resulting variability between samples and within transcripts.

Differential expression analysis

Quantification of gene expression levels (read counts per gene) was performed using QoRTs (v1.1.6; reference GRCh38.82; minMAPQ=50) (Hartley and Mullikin, 2015). Count data were imported into DESeq2 (v1.12.4) for quality assessment and differential expression analysis. Principal component analysis of rlog-transformed counts showed clear separation of cases and controls. Genes differentially expressed in PWS cases versus controls were identified from count data using DESeq2 (v1.12.4) with default parameters (Love et al., 2014). Ensembl Gene IDs with an official HUGO gene symbol (biomaRt, Ensembl Genes 87 (Smedley et al., 2015) and BH-adjusted $p < 0.25$ (unadjusted $p < 0.037$ with no automatic filtering) were reported as differentially expressed (DEGs; Table S1). Overlapping miRNAs were annotated by matching Ensembl Gene IDs to Entrez Gene IDs (org.Hs.eg.db v3.3.0 (Carlson, 2016). For comparison with other expression data sets, all Ensembl GeneIDs were included without filtering for HUGO gene symbol allowing for greater overlap of non-coding RNAs (Table S1). Visualisations and clustering were performed using rlog-transformed counts.

Comparing DEGs to independent gene expression data sets

DEGs were compared with independent gene expression data sets as follows: DEGs were compared with genes previously reported to be deregulated in PWS hypothalamus (Affymetrix Human Junction Array; HJAY) (Falaleeva et al., 2015). Matching was performed using stable reported Ensembl Gene IDs (HJAY probes mapped to hg18; taken from Supplementary Data 4 in (Falaleeva et al., 2015) and DEGs were cross-referenced with the FAL2015 data set (Table S1). We inspected individual genes reported in other studies using reported gene names (Burnett et al., 2017).

Cell types by inspection of cell marker expression data and by CIBERSORT

We investigated the cell type composition of the bulk tissue RNA-seq samples using two methods. First, we obtained a set of putative cell type-specific gene expression markers derived from purified neurons, astrocytes, microglia, oligodendrocytes and endothelial cells from mouse cortex (Zhang et al., 2014). For each of these cell types, we used the web tool:
http://web.stanford.edu/group/barres_lab/cgi-bin/enrich_cgi2.py to obtain the top 100 genes ranked by fold enrichment in that cell type (FPKM divided by the mean of FPKM values from all the other available cell types). We then inspected the overlap with DEGs (Figure 1D). As a second method, we estimated the proportion of specific cell types in the bulk tissue samples using CIBERSORT with sequencing profiles derived from human brain (Newman et al., 2015). Gene expression was quantified using the RSubRead package (Liao et al., 2014) using GENCODE release 25 (Harrow et al., 2012) as annotation. Counts were converted to fragments per kilobase of exon per million mapped reads (FPKM; (Trapnell et al., 2010) using length information from GENCODE and library size factors from DESeq2 (Love et al., 2014). 420 single-cell sequencing profiles from human brain (Darmanis et al., 2015) were aligned and quantified by the authors of (Yu and He, 2017), who used CIBERSORT (Newman et al., 2015) to create a signature matrix of genes that distinguish between 8 specific cell types. This matrix was then used to estimate cell-type proportions from the bulk tissue hypothalamus samples using CIBERSORT. Proportions of each cell type, if both significant, were compared between control and PWS samples with a t-test in R. Uncorrected p-values are reported for each cell type.

Transcript splicing and differential splicing analysis

To mitigate the observed 3’ bias, the quantification of transcript splicing and differential splicing analysis was performed on individual localised splicing events (rather than modelling differential exon usage for

the entire gene). In detail, quantification of transcript splicing was performed using SGSeq (v1.8.1) (Goldstein et al., 2016) by comparing to annotated transcripts (GRCH38.82). Read counts (*'countsVariant5pOr3p'*) were used as input for differential splicing analysis using DEXSeq (v1.20.2) (Anders et al., 2012) using the design formula ~ *sample + condition*exon* with SGSeq *'events'* and *'variants'* modelled as DEXSeq *'groups' and 'features'*, respectively.

Motif discovery

Motif discovery and motif similarity searches were performed using (MEME suite v4.11.3) (Bailey and Elkan, 1994). Motif discovery was performed using DREME suite (Bailey, 2011) for strand-specific motif discovery compared to shuffled sequences. Motif searches of putative retained introns were performed using the predicted retained introns including 250bp 5' and 3' flanking regions. Motifs were inspected for similarity to known RNA binding motifs using TOMTOM.

In silico prediction of SNORD116 gene targets

Genome-wide *in silico* prediction of SNORD116 targets was performed using snoTarget software (Bazeley et al., 2008) and RNA-cofold from the Vienna RNA package (http://www.tbi.univie.ac.at/RNA/) (Lorenz et al., 2011). All SNORD116 non-identical copies were used as queries against the human genome. Cuttoff of minimum free energy (MEF) <15 was applied.

Gene set enrichment analysis of DEGs

Enrichment statistics for curated pathways, Gene Ontology terms and keywords were calculated using DAVID v6.8 (Huang da et al., 2009a, b) and illustrated using Cytoscape (Shannon et al., 2003). Overlap statistics for MSigDB gene set collections C2 and H (MSigDB v5.2) were calculated using the GSEA/MSigDB 'Compute Overlaps' webtool (website v5.0) (Subramanian et al., 2005).

Ingenuity Pathway Analysis (IPA)

DEGs were analysed using Ingenuity Pathway Analysis software (Thermo Fisher Scientific, UK; Ingenuity IPA, Application Version 448560M, Content Version 36601845, Build: ing_narnia) using default parameters for Expression Analysis (Canonical Pathways, Upstream Regulator Analysis, and Regulator Effects) unless otherwise stated. The networks and functional analyses were generated using IPA (QIAGEN Inc., https://www.qiagenbioinformatics.com/products/ingenuity-pathway-analysis) (Kramer et al., 2014).

Cross-species comparison with Agrp and Pomc neuronal subtypes and response to food-deprivation

Reference gene sets for broad neuronal subtype classifications were derived from (Campbell et al., 2017). We defined **"AgRP neurons"** are as neuronal subtypes n12 (Agrp/Sst) and n13 (Agrp/Gm8773), **"POMC neurons "** as n14 (Pomc/Ttr), n15 (Pomc/Anxa2) and n21 (Pomc/Glipr1), and **"Other neurons"** as all other neuronal subtypes with available fold-change data (n1-n11, n16-n18, n20, n22-n32; as FDR values are not available) (Figure S2A-B). For each individual subtype, the top 100 genes by "Average" fold-change were obtained from "Suppl Table 4" of (Campbell et al., 2017). Then for each broad neuronal category (AgRP, Pomc, other neurons) the union of these top-ranked genes was taken as a reference gene set for that category (AgRP: 167 genes; POMC: 261 genes; Other neurons: 1589 genes). Known and putative neuronal subtype markers were inspected (Figure S2B) in order to verify that the reference gene lists used in this study approximate the patterns of expression markers reported in "Fig 3c" of (Campbell et al., 2017). Reference gene sets for fasting-response in AgRP neurons were obtained from (Henry et al., 2015) using a threshold of q < 0.05 (unless otherwise stated) to define differential expression between fasting conditions.

Validation of differentially expressed genes using quantitative RT-PCR

We tested a total of 30 gene transcripts and ncRNAs in the PWS region on chromosome 15 and a random subset of additional differentially expressed genes (DEGs) by quantitative reverse transcription a real-time PCR (qRT–PCR). Reverse transcription was performed using a RetroScript cDNA synthesis kit (Ambion, Thermo Scientific, UK). A total of 1 μg RNA was reverse transcribed using the RETROscript kit (Thermo Fischer Scientic, UK) according to the manufacturer's protocol. Real-time quantitative RT-PCR reactions were performed with 7900 HT Fast Real-Time PCR system (Applied Biosystems, Life Technologies) using the 2x SYBR Green PCR Master Mix (Life Technologies, Paisley, UK) and analyzed using ABI Prism 7000 SDS Software (Applied Biosystems, Foster City, CA). Relative mRNA levels of all genes were first normalized to the levels of GAPDH using the 2(-Delta Delta C (T)) method (Pfaffl et al. 2001), then normalized to the average of control levels. We used in-house designed gene/noncoding RNA-specific primers (available on request). In all cases expression was compared with that of GAPDH measured on the same sample in parallel on the same plate. Data was analysed for statistical

significance using unpaired nonparametric Mann-Whitney test and visualised using Graphpad Prism 6. Most of the core transcripts tested within the PWS region were found to be significantly downregulated in PWS cases versus controls (Figure S1E). In total, differential expression was confirmed in 89% (16/18) of tested genes.

Validation of differentially expressed genes by immunohistochemistry and *in situ* hybridization

Samples

Human hypothalamic tissue samples were obtained from the NICHD Brain & Tissue Bank, Maryland USA. Brains of 4 PWS (26 +/- 9 yrs) and 4 age-, gender-, ethnicity-, and PMI-matched controls (27 +/- 7 yrs) and Caucasian males were obtained from autopsies 6-29 h postmortem (details in Figure S1A). Tissue samples were immersion-fixed in 10% formalin, cryoprotected in 20% buffered sucrose for 48 h, and embedded in Tissue Tek and frozen. Coronal hypothalamic sections (20 µm-thick) were then mounted onto Superfrost Plus slides.

Immunohistochemistry (IHC)

Sections were processed for immunofluorescence using standard procedures (Bouret et al., 2004). The primary antibodies used for IHC were as follows: guinea-pig anti-oxytocin (1:10,000, Peninsula Laboratories), rabbit anti-GFAP (1:1,000, DAKO System), and rabbit anti-s100beta (1:200, Abcam). The primary antibodies were visualized with Alexa Fluor 488 donkey anti-guinea-pig IgGs or Alexa Fluor 488 goat anti-rabbit IgGs (1:200, ThermoFisher).

Fluorescent* in situ *hybridization

Sense and antisense digoxigenin-labeled riboprobes were generated from plasmids containing PCR fragments of BDNF and NTRK2 (generously provided by Dr. Baoji Xu, The Scripts Research Institute). Briefly, BDNF and NTRK2 plasmids were linearized using BamH1 and EcoR1, respectively, for the antisense probes and XhoI for the sense probes. Sense and antisense probes were then transcribed using T3 or T7 polymerases, respectively. Probes were purified using RNeasy MinElute Cleanup kit (Qiagen). Sections were incubated with Proteinase K (Promega). They were then incubated in triethanolamine (TEA), and in TEA containing glacial acid acetic. Sections were pre-hybridized in hybridization buffer containing deionized formamide, dextran sulfate, NaCl, Denhardt's Solution, Tris,

and EDTA. They were then hybridized with denatured probes (300 ng) overnight at 58°C. After washes in stringency solutions, sections were blocked in TNB solution (Roche) and incubated in a horseradish peroxidase-conjugated sheep anti-DIG antibody (1:400, Roche Applied Sciences). DIG was visualized using a TSA PLUS Biotin Kit (Perkin Elmer). Sections were first incubated in the Biotin Amplification Reagent (1:50), and then in streptavidin conjugated to cyanin 2 (1:200, Jackson Immunoresearch).

Image acquisition and analysis: Images were acquired using either a Zeiss LSM 710 confocal system equipped with a 20X or 40X objectives (IHC and ISH). The resulting image stack was analyzed using ImageJ analysis software (NIH). A series of contiguous sections stained with hematoxylin/eosin was used as a standard reference series. The atlas of the human hypothalamus of Baroncini et al (Baroncini et al., 2012) was also used to recognize the morphological limits of each nucleus. For illustration purposes, images from selected sections were captured using a Zeiss LSM 710 confocal system equipped with a 20X and 40X objectives.

Quantitative analysis of staining density and cell numbers

For the histological experiments, images of Bdnf, Ntrk2, GFAP and S100β, stainings, were aquired using a Zeiss LSM 710 confocal system equipped with a 20X objective.

For the quantitative analysis of staining density, each image was binarized to isolate staining from the background and to compensate for differences in fluorescence intensity. The integrated intensity, which reflects the total number of pixels in the binarized image, was then calculated for each picture. Image analysis was performed using Image J analysis software (NIH). The integrated density calculated for each image was used for statistical comparisons.

For the quantitative analysis of cell number, the numbers of Bdnf-, Ntrk2-, GFAP-, S100β-labeled cells in the VMH were manually counted. The number of cells counted in each image was used for statistical comparisons.

Hematoxylin and eosin staining

Series of adjacent 20-µm thick sections were cut from each block in the coronal plane and one was stained with hematoxylin and eosin (H&E). H&E images shown in Figures S3A,E are representative images of the VMH and PVN used for immunohistochemistry or in situ hybridization. We applied the recommendations in the neuroanatomical “MRI atlas of the Human Hypothalamis”, published by Baroncini et al ((Baroncini et al., 2012), p.168-180).

Measurements of plasma BDNF

The Medical Ethics Committee of Erasmus University Medical Center / Sophia Children's Hospital, Rotterdam approved the studies. Written informed consent was obtained from parents and from children older than 12 years; assent was obtained in children younger than 12 years of age. We included 25 children and 10 young adults with a genetically confirmed diagnosis of Prader Willi Syndrome by a positive methylation test. All subjects were participating in the Dutch PWS studies coordinated by the Dutch Growth Research Foundation and were treated with growth hormone (GH; ~0.033 mg/kg/day). In addition, 8 patients had been sampled before GH treatment. As there was no difference in BDNF levels with/without GH, this data was included. Obese controls from the Genetics of Obesity Study (GOOS; www.goos.org.uk) were included for comparison after ethical committee approval (Cambridge REC 03/103) and after informed consent. Blood samples were collected in lithium heparin tubes after a 12h overnight fast, centrifuged within 30 minutes and then immediately stored at -80 C until assayed. Frozen samples were assayed for BDNF using the MesoScale discovery platform (R&D System reagents).

Cellular studies

Cell Culture Maintenance

SH-SY5Y (ATCC® CRL-2266) cell line were grown in Dulbecco's Modified Eagle's Medium: Nutrient Mixture F-12 (DMEM/F-12; Fisher Scientific) supplemented with Non-Essential Amino Acids (NEAA; Thermo Fisher Scientific) and 10% vol/vol fetal bovine serum (FBS; Thermo Fisher Scientific). Cultures were grown in TC75cm2 flasks and maintained at 37°C and 5% CO2 in a humidified chamber.

SNORD116 cluster deletion using CRISPR-Cas 9

We applied a cloning-free CRISPR protocol relying on gBlocks (gene fragments) encoding FE-modified sgRNAs promoting enhanced stability (Arbab et al., 2015; Chen et al., 2013). Two gBlocks carrying the guide RNAs (sg1: 5'-CCACTCTCATTCAGCACGT-3' sg2:5'-AGCCATCCATAAGTTATCT-3') were predicted to target the sequence flanking the SNORD116 cluster on chr15q11.2 using the Zhang Lab server (www.crispr.mit.edu) with minimal off-target binding in genes and were synthetized by ITD Inc, (USA).

All nucleofections were performed using the CA-137 program on a 4D-Nucleofector (Lonza). Each nucleofection reaction was composed of 1 x 106 SH-SY5Y cells, 1.5µg of sgRNA1, 1.5µg sgRNA 2, 5µg Cas9 plasmid (GFP-expressing plasmid PX458; Addgene) and 100µl of nucleofection solution (SF Cell Line 4D-Nucleofector X Kit, Lonza). Two days post-nucleofection, cells were FACS sorted and plated into 96-well plates at a density of 1 cell per well. Individual colonies were expanded, DNA extracted using QuickExtract DNA extraction solution (EpiCentre, UK) and screened for successful editing using conventional PCR (primers available on request). Sanger sequencing of the PCR products was performed with BigDye v3.0 biochemistry (Thermo Fisher Scientific) on a capillary sequencer ABI3730 (Applied Biosystems, UK) according to the manufacturer instructions.

Neuronal Differentiation

We used a classical protocol developed by (Encinas et al., 2000) to differentiate SH-SY5Y cells into neurons with Retinoic acid (RA). The cultures were plated at a density of 2 x 104 cells per well of a 6-well plate or 35mm dish. For immunofluorescence, cells were plated onto 22mm glass coverslips (Scientific Laboratory Supplies). At day 0 (one day after plating), media was changed to differentiation media composed of basal DMEM/F-12 supplemented with 1% NEAA, 1% vol/vol FBS and 10µM retinoic acid (Sigma). For BDNF 'rescue' differentiation assays, media was changed to differentiation media further supplemented with 50ng/ml BDNF (dissolved in 0.1% BSA-H2O; Tocris). Fresh differentiation media, with the appropriate supplements, was used to replace media every two days until day 7. On day 7 of differentiation, cells were fixed on coverslips (100% cold methanol incubation for 10 minutes). After fixation, coverslips were washed and covered with PBS, then stored at 4°C for immunofluorescence. Fixed cells were permeabilised in 0.1% Triton X100-PBS for 15 minutes. To block, cells were incubated in 2% BSA in PBS for 1 hour on an orbital shaker. Finally, coverslips were incubated at 4°C overnight in a mix of 1µg/ml anti-beta III tubulin primary antibody (ab18207, Abcam), 1% BSA and 0.1% Triton X100 in PBS. Coverslips were incubated for 1 hour in a mix of 1µg/ml AlexaFluor488 secondary antibody (ab150081, Abcam), 1:100,000 HCS CellMask Deep Red (Thermo Fisher Scientific) and 1µg/ml DAPI (Sigma) in PBS. Images were taken at 20X using an INCA 2200 (GE Healthcare Life Sciences); a minimum of 15 randomly selected fields were visualized per cell type, per replicate. The number of differentiated cells was counted and expressed as a proportion of total cells observed. Neurons were defined as possessing small cell bodies, with a high nuclear:cytoplasmic ratio, and at least one neurite with a length that was greater than or equal to cell body diameter. A minimum of 100 cells were analysed for each cell line per replicate, from a minimum of 6 fields.

Proliferation assay

EdU (5-ethynyl-2'-deoxyuridine) labelling of newly synthesised DNA was performed with Click-iT® EdU Alexa Fluor® 488 Imaging Kit (Thermo Fisher Scientific), according to manufacturer's instructions. Briefly, cells were treated with retinoic acid with/without BDNF, as previously described. On Day 7, half of the media volume was removed and replaced with 10μM EdU media. Cultures were incubated in EdU for 4 hours at 37˚C, before fixation. Cells were permeabilised with 0.5% Triton-X100 in PBS for 20 mins, before 30 mins incubation with Click-iT reaction mix. Cells were stained with DAPI and imaged on an LSM710 confocal microscope (Zeiss, UK) with a 20X objective. The number of proliferating (EdU positive) cells was counted from a minimum of 150 cells and 4 randomly selected fields.

Cell survival and apoptosis assays

On day 7 of apoptosis assays, cells were incubated with FITC Annexin V antibody (BD Biosciences) for 15 minutes, after which 5μg/ml propidium iodide (BD Biosciences) was added. Analysis was conducted on the NovoCyte flow cytometer system (Acea Biosciences).

Statistical analysis

All experiments were conducted with 3-6 separate experiments (each including 3 technical replicates). Results were analysed using GraphPad Prism 6 and statistical significance measured applying unpaired Student t test was used to generate statistical analysis. P values < 0.05 were considered statistically significant. All results are expressed as mean values ± SEM of at least three independent experiments.

SUPPLEMENTAL REFERENCES

Anders, S., Reyes, A., and Huber, W. (2012). Detecting differential usage of exons from RNA-seq data. Genome Res *22*, 2008-2017.

Arbab, M., Srinivasan, S., Hashimoto, T., Geijsen, N., and Sherwood, R.I. (2015). Cloning-free CRISPR. Stem cell reports *5*, 908-917.

Bailey, T.L. (2011). DREME: motif discovery in transcription factor ChIP-seq data. Bioinformatics *27*, 1653-1659.

Bailey, T.L., and Elkan, C. (1994). Fitting a mixture model by expectation maximization to discover motifs in biopolymers. Proc Int Conf Intell Syst Mol Biol *2*, 28-36.

Baroncini, M., Jissendi, P., Balland, E., Besson, P., Pruvo, J.P., Francke, J.P., Dewailly, D., Blond, S., and Prevot, V. (2012). MRI atlas of the human hypothalamus. NeuroImage *59*, 168-180.

Bazeley, P.S., Shepelev, V., Talebizadeh, Z., Butler, M.G., Fedorova, L., Filatov, V., and Fedorov, A. (2008). snoTARGET shows that human orphan snoRNA targets locate close to alternative splice junctions. Gene *408*, 172-179.

Bouret, S.G., Draper, S.J., and Simerly, R.B. (2004). Trophic Action of Leptin on Hypothalamic Neurons That Regulate Feeding. Science *304*, 108-110.

Burnett, L.C., LeDuc, C.A., Sulsona, C.R., Paull, D., Rausch, R., Eddiry, S., Carli, J.F., Morabito, M.V., Skowronski, A.A., Hubner, G.*, et al.* (2017). Deficiency in prohormone convertase PC1 impairs prohormone processing in Prader-Willi syndrome. J Clin Invest *127*, 293-305.

Campbell, J.N., Macosko, E.Z., Fenselau, H., Pers, T.H., Lyubetskaya, A., Tenen, D., Goldman, M., Verstegen, A.M., Resch, J.M., McCarroll, S.A.*, et al.* (2017). A molecular census of arcuate hypothalamus and median eminence cell types. Nat Neurosci *20*, 484-496.

Carlson, M. (2016). org.Hs.eg.db: Genome wide annotation for Human.

Chen, B., Gilbert, L.A., Cimini, B.A., Schnitzbauer, J., Zhang, W., Li, G.W., Park, J., Blackburn, E.H., Weissman, J.S., Qi, L.S.*, et al.* (2013). Dynamic imaging of genomic loci in living human cells by an optimized CRISPR/Cas system. Cell *155*, 1479-1491.

Darmanis, S., Sloan, S.A., Zhang, Y., Enge, M., Caneda, C., Shuer, L.M., Hayden Gephart, M.G., Barres, B.A., and Quake, S.R. (2015). A survey of human brain transcriptome diversity at the single cell level. Proc Natl Acad Sci U S A *112*, 7285-7290.

Dobin, A., Davis, C.A., Schlesinger, F., Drenkow, J., Zaleski, C., Jha, S., Batut, P., Chaisson, M., and Gingeras, T.R. (2013). STAR: ultrafast universal RNA-seq aligner. Bioinformatics *29*, 15-21.

Encinas, M., Iglesias, M., Liu, Y., Wang, H., Muhaisen, A., Cena, V., Gallego, C., and Comella, J.X. (2000). Sequential treatment of SH-SY5Y cells with retinoic acid and brain-derived neurotrophic factor gives rise to fully differentiated, neurotrophic factor-dependent, human neuron-like cells. J Neurochem *75*, 991-1003.

Falaleeva, M., Surface, J., Shen, M., de la Grange, P., and Stamm, S. (2015). SNORD116 and SNORD115 change expression of multiple genes and modify each other's activity. Gene *572*, 266-273.

Goldstein, L.D., Cao, Y., Pau, G., Lawrence, M., Wu, T.D., Seshagiri, S., and Gentleman, R. (2016). Prediction and Quantification of Splice Events from RNA-Seq Data. PLOS ONE *11*, e0156132.

Harrow, J., Frankish, A., Gonzalez, J.M., Tapanari, E., Diekhans, M., Kokocinski, F., Aken, B.L., Barrell, D., Zadissa, A., Searle, S.*, et al.* (2012). GENCODE: the reference human genome annotation for The ENCODE Project. Genome Res *22*, 1760-1774.

Hartley, S.W., and Mullikin, J.C. (2015). QoRTs: a comprehensive toolset for quality control and data processing of RNA-Seq experiments. BMC Bioinformatics *16*, 224.

Henry, F.E., Sugino, K., Tozer, A., Branco, T., and Sternson, S.M. (2015). Cell type-specific transcriptomics of hypothalamic energy-sensing neuron responses to weight-loss. eLife *4*.

Huang da, W., Sherman, B.T., and Lempicki, R.A. (2009a). Bioinformatics enrichment tools: paths toward the comprehensive functional analysis of large gene lists. Nucleic Acids Res *37*, 1-13.

Huang da, W., Sherman, B.T., and Lempicki, R.A. (2009b). Systematic and integrative analysis of large gene lists using DAVID bioinformatics resources. Nat Protoc *4*, 44-57.

Kramer, A., Green, J., Pollard, J., Jr., and Tugendreich, S. (2014). Causal analysis approaches in Ingenuity Pathway Analysis. Bioinformatics *30*, 523-530.

Liao, Y., Smyth, G.K., and Shi, W. (2014). featureCounts: an efficient general purpose program for assigning sequence reads to genomic features. Bioinformatics *30*, 923-930.

Lorenz, R., Bernhart, S.H., Honer Zu Siederdissen, C., Tafer, H., Flamm, C., Stadler, P.F., and Hofacker, I.L. (2011). ViennaRNA Package 2.0. Algorithms for molecular biology : AMB *6*, 26.

Love, M.I., Huber, W., and Anders, S. (2014). Moderated estimation of fold change and dispersion for RNA-seq data with DESeq2. Genome Biol *15*, 550.

Newman, A.M., Liu, C.L., Green, M.R., Gentles, A.J., Feng, W., Xu, Y., Hoang, C.D., Diehn, M., and Alizadeh, A.A. (2015). Robust enumeration of cell subsets from tissue expression profiles. Nat Methods *12*, 453-457.

Parikshak, N.N., Swarup, V., Belgard, T.G., Irimia, M., Ramaswami, G., Gandal, M.J., Hartl, C., Leppa, V., Ubieta, L.T., Huang, J.*, et al.* (2016). Genome-wide changes in lncRNA, splicing, and regional gene expression patterns in autism. Nature *540*, 423-427.

Shannon, P., Markiel, A., Ozier, O., Baliga, N.S., Wang, J.T., Ramage, D., Amin, N., Schwikowski, B., and Ideker, T. (2003). Cytoscape: a software environment for integrated models of biomolecular interaction networks. Genome Res *13*, 2498-2504.

Smedley, D., Haider, S., Durinck, S., Pandini, L., Provero, P., Allen, J., Arnaiz, O., Awedh, M.H., Baldock, R., Barbiera, G.*, et al.* (2015). The BioMart community portal: an innovative alternative to large, centralized data repositories. Nucleic Acids Res *43*, W589-598.

Subramanian, A., Tamayo, P., Mootha, V.K., Mukherjee, S., Ebert, B.L., Gillette, M.A., Paulovich, A., Pomeroy, S.L., Golub, T.R., Lander, E.S.*, et al.* (2005). Gene set enrichment analysis: a knowledge-based approach for interpreting genome-wide expression profiles. Proc Natl Acad Sci U S A *102*, 15545-15550.

Trapnell, C., Williams, B.A., Pertea, G., Mortazavi, A., Kwan, G., van Baren, M.J., Salzberg, S.L., Wold, B.J., and Pachter, L. (2010). Transcript assembly and quantification by RNA-Seq reveals unannotated transcripts and isoform switching during cell differentiation. Nat Biotechnol *28*, 511-515.

Yu, Q., and He, Z. (2017). Comprehensive investigation of temporal and autism-associated cell type composition-dependent and independent gene expression changes in human brains. Sci Rep *7*, 4121.

Zhang, Y., Chen, K., Sloan, S.A., Bennett, M.L., Scholze, A.R., O'Keeffe, S., Phatnani, H.P., Guarnieri, P., Caneda, C., Ruderisch, N.*, et al.* (2014). An RNA-sequencing transcriptome and splicing database of glia, neurons, and vascular cells of the cerebral cortex. J Neurosci *34*, 11929-11947.

A

ID	Diagnosis	UMB ID	Age (yr)	BMI (kg/m^2)	Ethnicity	PMI (h)	RIN	Properly paired reads
1	Control	1671	0.8	17.6	AA	18	7.0	74257798 (95.66%)
1	PWS	5441	0.9	17.3	AA	18	5.1	77004500 (94.96%)
2	Control	4781	46.0	27.4	C	17	6.5	76438058 (95.32%)
2	PWS	5685	45.4	--	C	6	6.9	74372578 (95.41%)
3	Control	5185	22.0	20.0	C	26	6.4	54105638 (94.89%)
3	PWS	5726	22.5	25.3	C	29	4.4	85445062 (95.66%)
4	Control	5659	17.9	25.5	C	10	5.8	73996056 (95.59%)
4	PWS	5731	17.2	18.3	C	8	6.4	86020934 (95.67%)
IHC	Control	1226	23.2	27.7	C	21		
	Control	1322	16.6	24.5	C	25		
	Control	5654	19.7	20.0	C	18		
	Control	5668	47.0	27.1	C	23		
	PWS	5324	18.8	54.7	C	22		
	PWS*	5685	45.4	--	C	6		
	PWS*	5731	17.2	18.3	C	8		

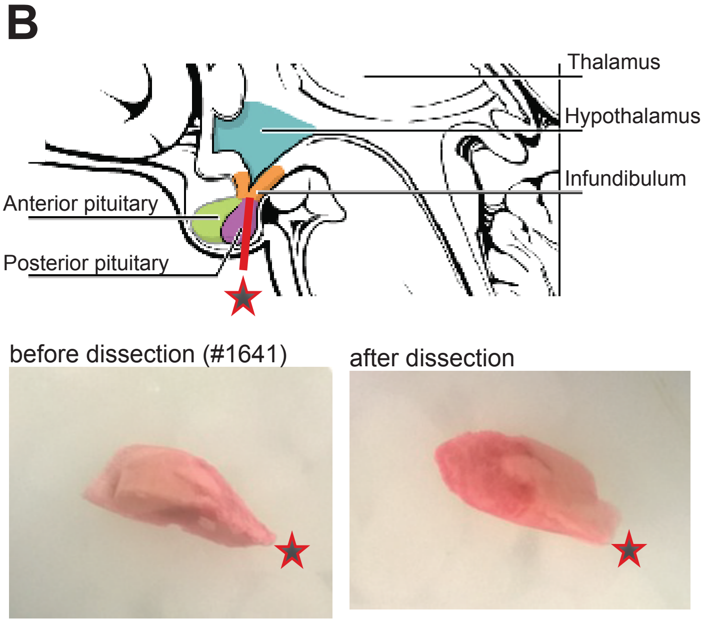

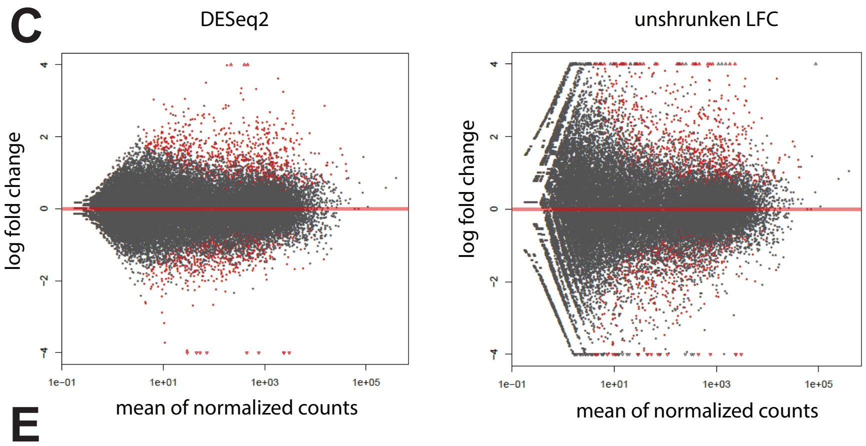

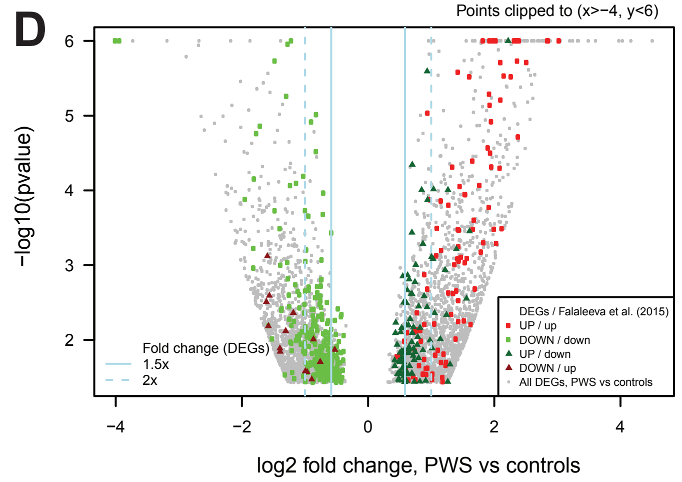

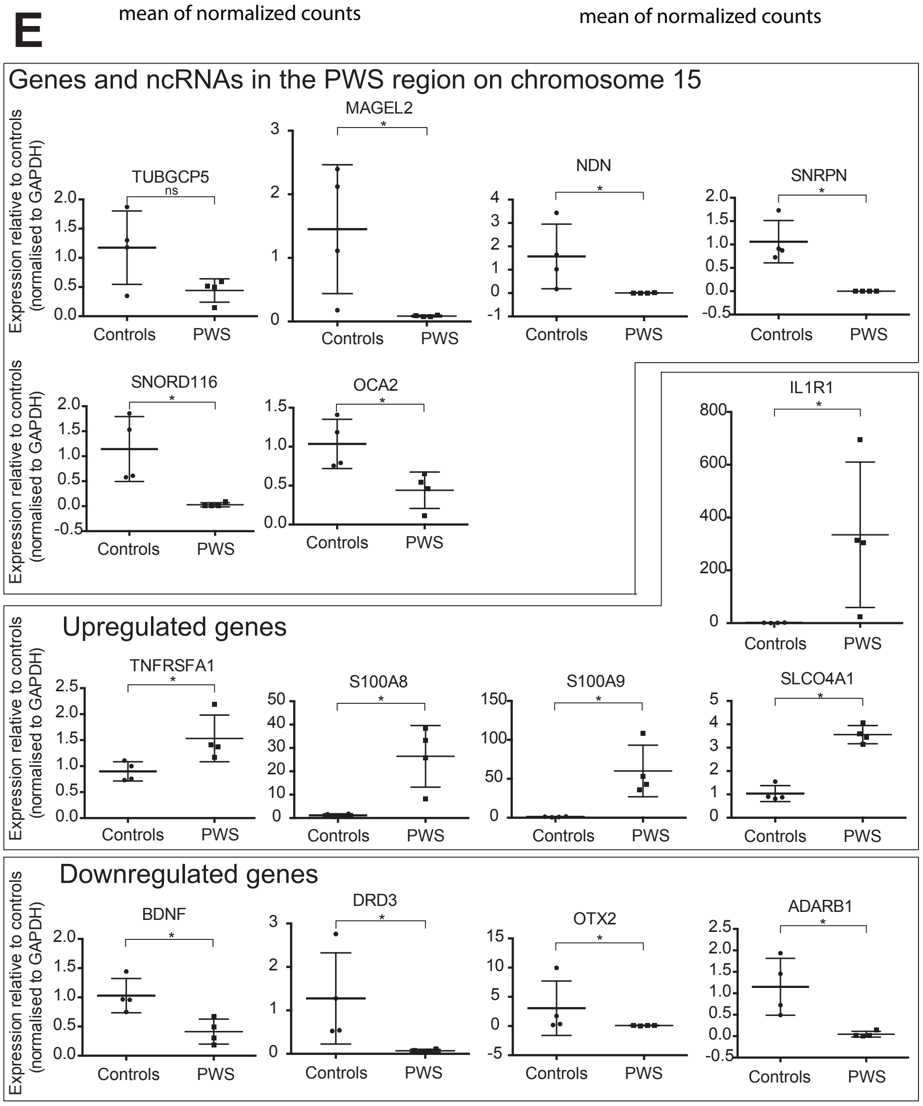

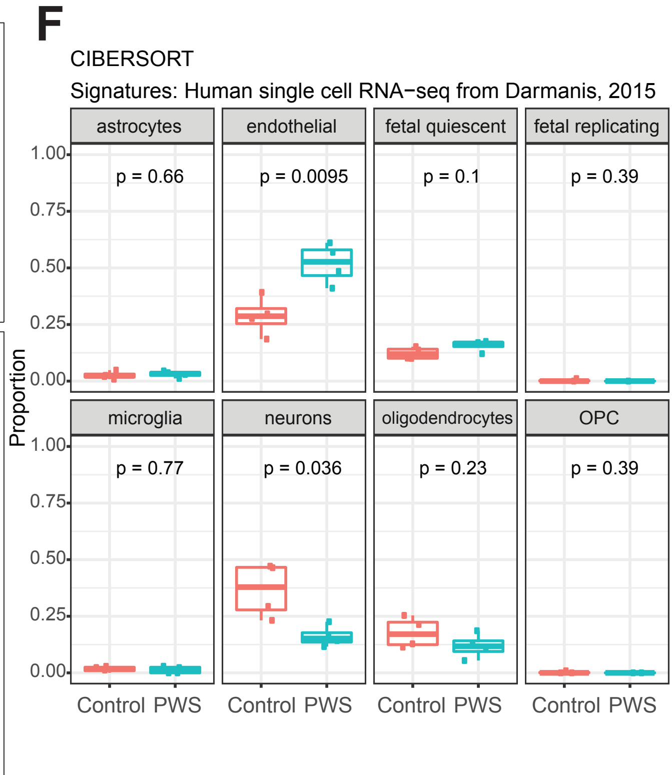

Supplemental Figure 1. RNA-seq of hypothalamic samples. Related to Figure 1 and Supplemental Experimental Methods. Full legend on the next page.

Supplemental Figure 1. RNA-seq of hypothalamic samples. Related to Fig 1 and Supplemental Experimental Methods.

(A) Patient characteristics and sample information for RNA-seq (top) and immunohistochemical analysis (IHC, bottom). ID, case-control pair identifier for RNA-seq; UMB ID, University of Maryland Brain and Tissue Bank sample identifier. Ethnicity: AA, African American; C, Caucasian. PMI, Post-mortem interval; RIN, RNA integrity number; *, IHC samples also in RNA-seq study.

(B) Dissection protocol for human hypothalamic RNA-seq of one-third left hemisphere hypothalamic volume. The shape of the median eminence towards the infundibulum/pituitary was used as an anatomical marker (indicated with star) and dissection on the opposite side was performed, removing approximately one-third volume and containing mostly posterior and lateral hypothalamus. Sample #1641 is shown before and after dissection. *[Illustration adapted from Anatomy & Physiology, Connexions Web site. http://cnx.org/content/col11496/1.6/, Jun 19, 2013; via Wikimedia Commons, CC BY 3.0].*

(C)-(D) MA plots and volcano plots of DESeq2 results. (C) MA plots showing M ('log ratio', log fold change) versus A ('mean average', mean of normalized counts). Plots show DESeq2 results (left, with shrinkage) and unshrunken log fold change (LFC) (right). (D) Volcano plot of DEGs. DEGs which were also reported to be dysregulated in FAL2015 are indicated (legend inset).

(E) Gene expression of individual genes measured by quantitative reverse transcription a real-time PCR (qRT–PCR). Genes in the PWS region on chromosome 15 (top), and a selection of top genes upregulated (middle) or downregulated in PWS (bottom). Relative mRNA levels normalized to GAPDH are presented, relative to control levels. Statistical significance was established with unpaired nonparametric Mann-Whitney U-test, p-value < 0.05 is denoted with asterisk, and non significant as 'ns'.

(F) Cell type proportions estimated using CIBERSORT. Proportions of each cell type in PWS and in control samples were estimated using CIBSESORT (details in Supplementary Methods) and compared between control and PWS samples (t-test, two-tailed).

A

Gene Set Name	# Genes in Gene Set (K)	Description	# Genes in Overlap (k)	k/K	p-value	FDR q-value
**DEGs DOWN**						
BLALOCK_ALZHEIMERS_DISEASE_DN	1237	Genes down-regulated in brain from patients with Alzheimer's disease.	134	0.11	2E-47	3E-44
KIM_ALL_DISORDERS_CALB1_CORR_UP	548	Genes whose expression significantly and positively correlated with the density of CALB1-positive [GeneID=793] GABAergic interneurons in the BA9 brain region across all subjects with psychiatric disorders.	84	0.15	4E-41	4E-38
MEISSNER_BRAIN_HCP_WITH_H3K4ME3_AND_H3K27ME3	1069	Genes with high-CpG-density promoters (HCP) bearing histone H3 dimethylation at K4 (H3K4me2) and trimethylation at K27 (H3K27me3) in brain.	110	0.1	8E-37	5E-34
REACTOME_NEURONAL_SYSTEM	279	Genes involved in Neuronal System	46	0.16	2E-24	8E-22
KEGG_NEUROACTIVE_LIGAND_RECEPTOR_INTERACTION	272	Neuroactive ligand-receptor interaction	41	0.15	2E-20	6E-18
KIM_BIPOLAR_DISORDER_OLIGODENDROCYTE_DENSITY_CORR_UF	682	Genes whose expression significantly and positively correlated with oligodendrocyte density in layer VI of BA9 brain region in patients with bipolar disorder.	61	0.09	8E-18	2E-15
KIM_ALL_DISORDERS_OLIGODENDROCYTE_NUMBER_CORR_UP	756	Genes whose expression was significantly and positively correlated with the number of perineuronal oligodendrocytes in the layer III of BA9 brain region.	62	0.08	3E-16	5E-14
LU_AGING_BRAIN_DN	153	Age down-regulated genes in the human frontal cortex.	23	0.15	5E-12	5E-10
LEIN_LOCALIZED_TO_PROXIMAL_DENDRITES	37	Transcripts showing subcellular localization only to proximal dendrites in the adult mouse brain.	12	0.32	5E-11	4E-09
REACTOME_NEUROTRANSMITTER_RELEASE_CYCLE	34	Genes involved in Neurotransmitter Release Cycle	11	0.32	3E-10	2E-08
**DEGs UP**						
BLALOCK_ALZHEIMERS_DISEASE_UP	1691	Genes up-regulated in brain from patients with Alzheimer's disease.	281	0.17	3E-133	1E-129
HALLMARK_TNFA_SIGNALING_VIA_NFKB	200	Genes regulated by NF-kB in response to TNF [GeneID=7124].	83	0.42	2E-73	4E-70
KEGG_RIBOSOME	88	Ribosome	54	0.61	2E-60	2E-57
CHEN_METABOLIC_SYNDROM_NETWORK	1210	Genes forming the macrophage-enriched metabolic network (MEMN) claimed to have a causal relationship with the metabolic syndrom traits.	157	0.13	2E-57	2E-54

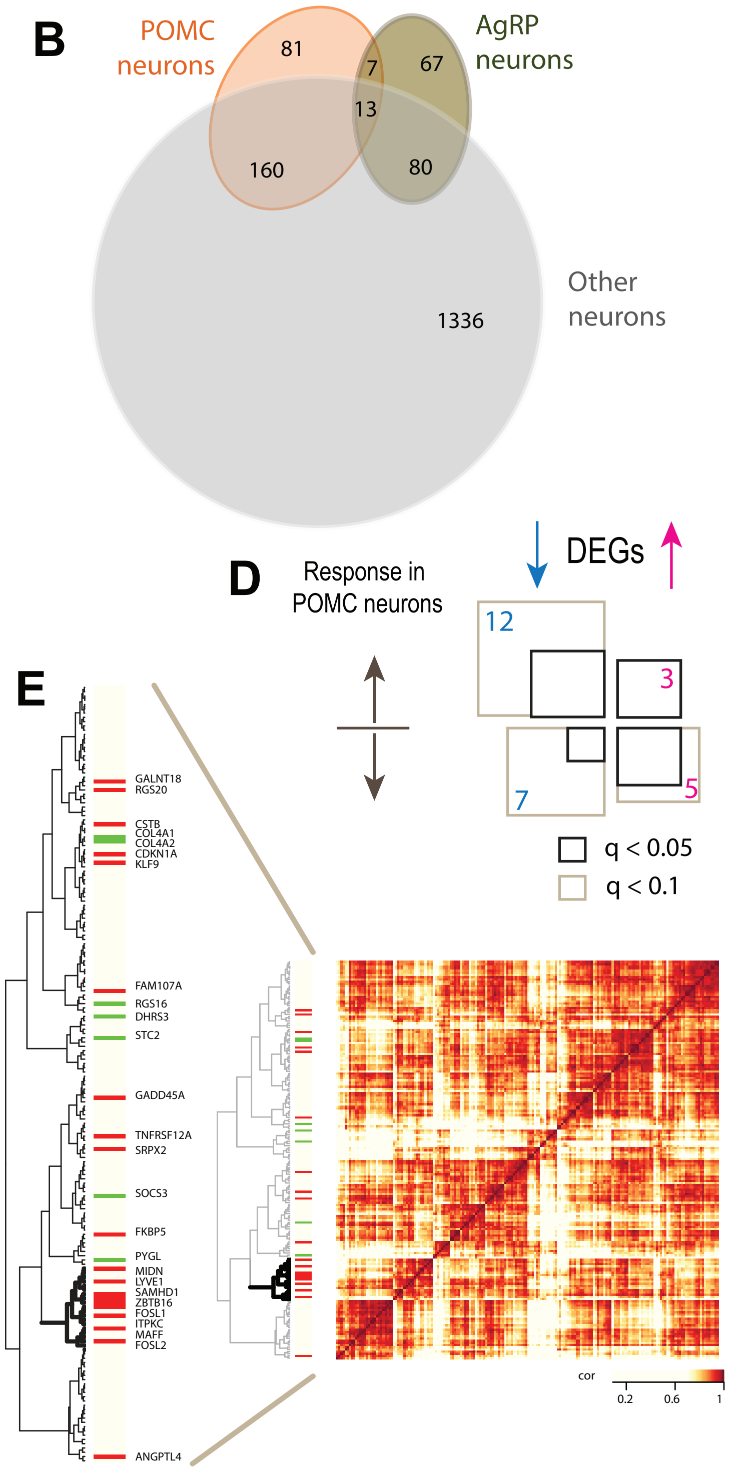

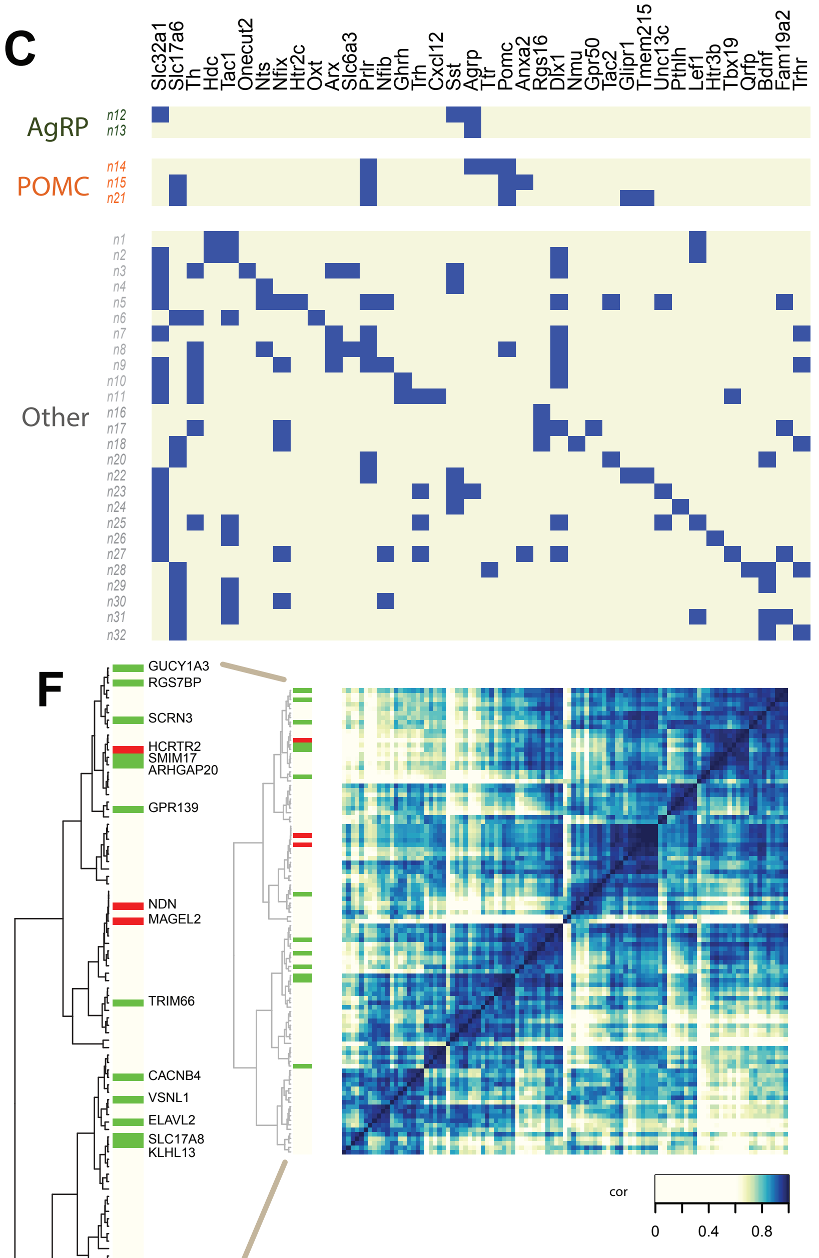

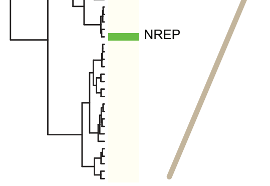

Supplemental Figure 2. DEGs and co-expressed gene modules overlap with neuronal subtypes and fasting-response. Related to Figure 2 and Supplemental Experimental Methods. Full legend on the next page.

Supplemental Figure 2. DEGs and co-expressed gene modules overlap with neuronal subtypes and fasting-response. Related to Figure 2 and Supplemental Experimental Methods.

(A) Overlap analysis of DEGs down (top) and up (bottom) in PWS compared to controls. Illustrative gene sets among the top 100 gene sets by enrichment (FDR) are shown (MSigDB; see Supplemental Experimental Methods).

(B)-(C) Reference gene sets for broad neuronal subtype classifications and their overlaps, based on Campbell et al 2017. (A) Venn diagram illustrates the relative proportions of the gene sets and provides a reference for inspections of DEGs (Figure 3a, inset). The different sizes of the gene sets reflect the definitions of “AgRP neurons”, “Pomc neurons” and “Other neurons” used in this study; see Supplemental Experimental Methods. (B) Inspection of known and putative neuronal subtype markers, verifying that the reference gene lists used in this study approximate the patterns of expression markers reported in (Campbell et al 2017 Fig 3c). Blue indicates that the gene is part of the reference gene set for the respective neuronal cell type.

(D) The direction of differential expression is shown for DEGs (PWS versus control) previously reported to be differentially expressed in Pomc neurons in fasted versus fed state (Henry et al. 2015).

(E) Hierarchical clustering of DEGs up in PWS with $q < 0.01$. Heatmap illustrates pairwise gene-gene correlation clustering (Pearson correlation, distance=1-cor, Ward clustering). Row sidebar displays the overlap with genes previously reported up- (red) or down-regulated (green) in AgRP neurons in fasted versus fed state ($q < 0.05$ in [Henry et al. 2015].

(F) Hierarchical clustering of DEGs down in PWS with $q < 0.02$. Row sidebar (left) as for (E).

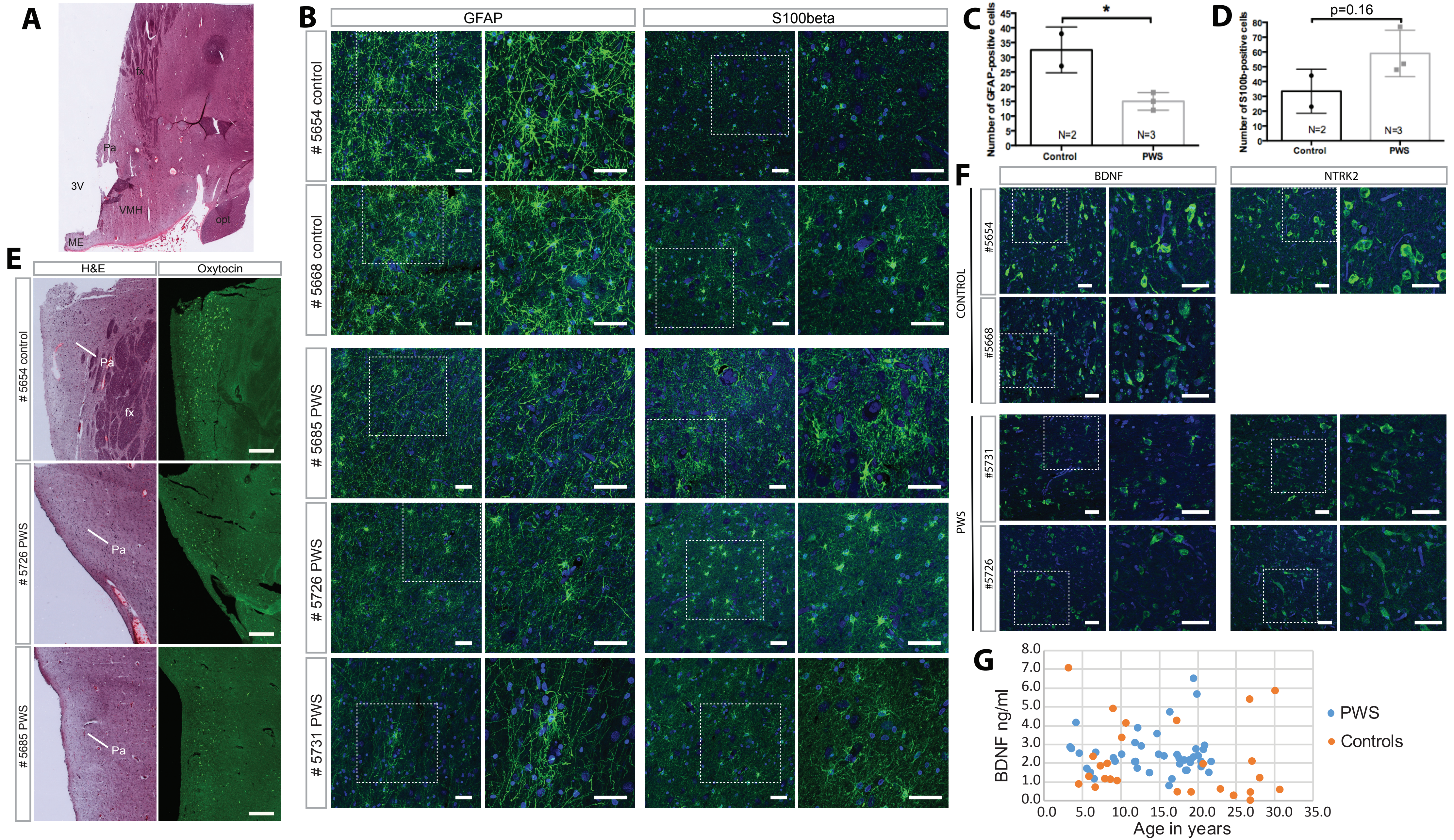

Supplemental Figure 3. Increase of S100b and reduction of GFAP, oxytocin, BDNF and NTRK2 in PWS hypothalamic tissue. Related to Figure 3. (A)-(D) GFAP and S100B reactivity in ventromedial hypothalamus (VMH) of controls and PWS samples. (A) Haematoxylin-eosin (H&E) staining of a representative sample. (B) GFAP and S100B expression; all images are shown in duplicate for two magnifications, 20x and 40x; scale bars are 50 microns. (C) Quantification of the number of GFAP-positive cells. (D) Quantification of the number of S100B-positive cells. The number of images used for the quantification is shown (*, p-value <0.05). **(E)** Oxytocin reactivity in paraventricular nucleus of the hypothalamus of controls and PWS samples. H&E staining of each sample is shown alongside the confocal images. Scale bars are 400 microns. (**F**) BDNF and NTRK2 reactivity in ventromedial hypothalamus (VMH) of controls and PWS samples; images are shown in duplicate at two magnifications, 20x and 40x; scale bars are 50 microns. **(G)** Plasma BDNF levels were measured in 43 children and adults with genetically proven PWS and 27 BMI matched controls in whom known genetic causes of obesity had been excluded. Abbreviations: 3V (third ventricle), fx (fornix), opt (optic tract), ME (median eminence), Pa (paraventricular nucleus).

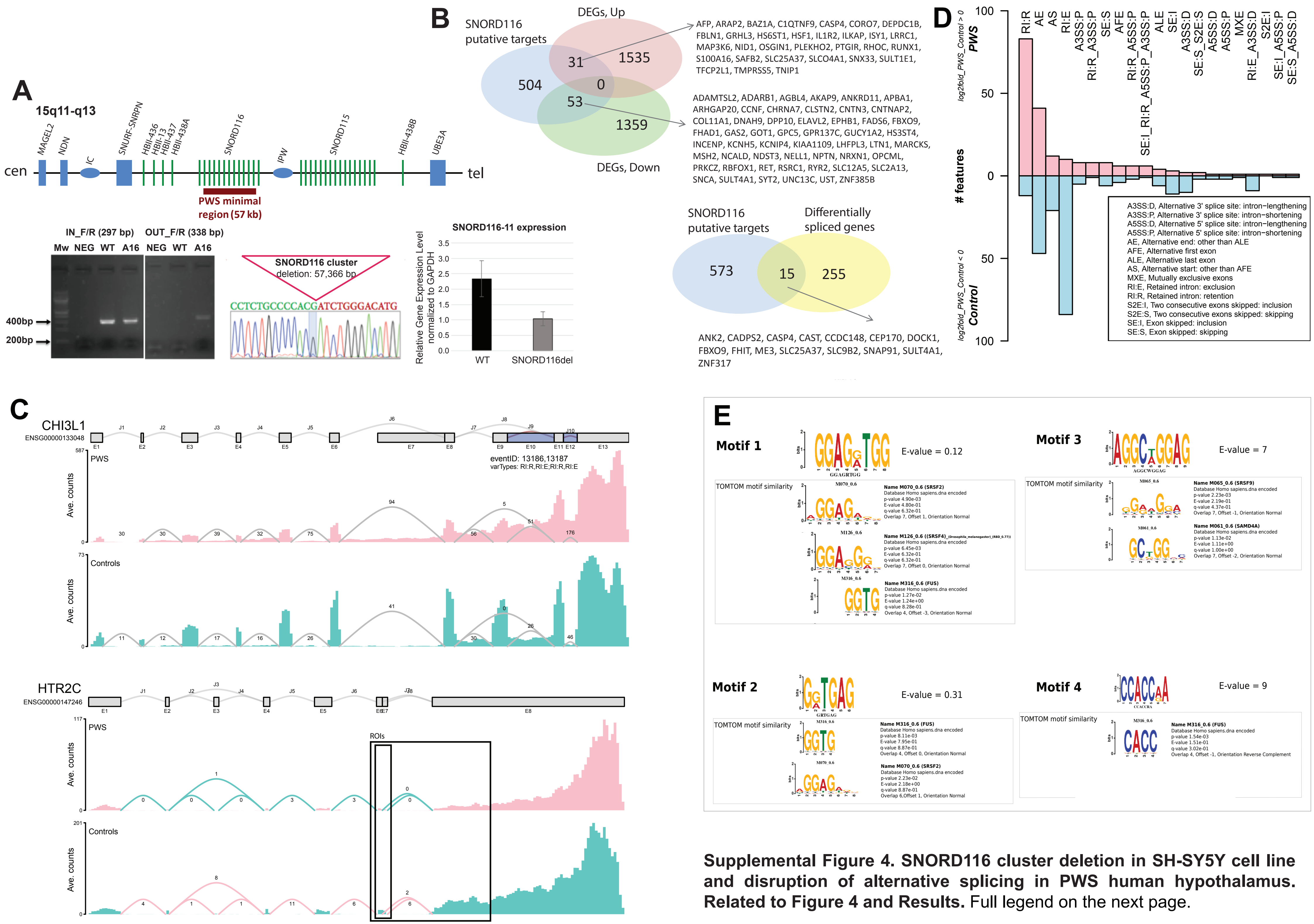

Supplemental Figure 4. SNORD116 cluster deletion in SH-SY5Y cell line and disruption of alternative splicing in PWS human hypothalamus. Related to Figure 4 and Results. Full legend on the next page.

Supplemental Figure 4. SNORD116 cluster deletion in SH-SY5Y cell line and disruption of alternative splicing in PWS human hypothalamus. Related to Figure 4 and Results.

(A) CRISPR/Cas9-mediated SNORD116 deletion from the PWS genomic locus on chromosome 15q11 **(top)** and confirmation by qPCR and Sanger sequencing. For the PCR screening strategy, two primer pairs outside (OUT) and inside (IN) the deleted segment were used in combination to screen DNA from individually picked and expanded colonies derived from single FACS-sorted cells. The gel image for the clone A16 used in this study is depicted **(left)** showing that PCR is successful for the IN and OUT PCR products. Deletion of SNORD116 was confirmed by Sanger sequencing of the junction fragment (OUT PCR product), where the sequences show joined ends of the targeted single-guide RNA sites (sgRNA1, sgRNA2) **(centre)**. Confirmation of deletion by qPCR measurement of SNORD116-11 expression is shown **(right)**. In addition we tested expression of SNORD116 copies -2, -3, -21, -29 and detected identical levels of 50% reduction, whereas neighbouring SNORD115-5 remained at similar levels between WT and SNORD116del cells (not shown).

(B) Overlap of predicted SNORD116 gene targets with DEGs (left) and with genes containing predicted differential splicing events (right).

(C) Illustrative splice graphs with average raw read counts, shown for CHI3L1 (top) and serotonin 2c receptor, HTR2C (bottom). Each gene displays splice graphs (upper, grey) and histograms of average raw counts for PWS (middle, pink) and controls (lower,turquoise) aligned to transcript features (E1, E2, etc.). Arced lines indicate the number of reads spanning the corresponding feature in the splice graph (J1, J2, etc.). Differential splicing events are indicated in the splice graph (purple); related to Supplemental Table 11. Boxes (’ROIs’) indicate regions-of-interest for inspection of alternative splicing.

(D) Barplot of differential splicing events (FDR < 0.25); related to Supplemental Table 11.

(E) DREME motif search in putative retained introns and flanking regions (+250bp) and TOMTOM motif similarity search.
